# Apelin: A putative novel predictive biomarker for bevacizumab response in colorectal cancer

**DOI:** 10.18632/oncotarget.17306

**Published:** 2017-04-21

**Authors:** Linda Zuurbier, Arman Rahman, Martijn Cordes, Jennifer Scheick, Tse J. Wong, François Rustenburg, Jesu Christopher Joseph, Peter Dynoodt, Rory Casey, Paul Drillenburg, Michael Gerhards, Ana Barat, Rut Klinger, Bozena Fender, Darran P. O’Connor, Johannes Betge, Matthias P. Ebert, Timo Gaiser, Jochen H. M. Prehn, Arjan W. Griffioen, Nicole C. T. van Grieken, Bauke Ylstra, Annette T. Byrne, Laurens G. van der Flier, William M. Gallagher, Ruben Postel

**Affiliations:** ^1^ SomantiX BV, Utrecht, The Netherlands; ^2^ OncoMark Ltd, Dublin, Ireland; ^3^ Department of Pathology, VU University Medical Center, Amsterdam, The Netherlands; ^4^ Department of Medical Oncology, Angiogenesis Laboratory, VU University Medical Center, Amsterdam, The Netherlands; ^5^ Department of Pathology, Onze Lieve Vrouwe Gasthuis, Amsterdam, The Netherlands; ^6^ Department of Physiology & Medical Physics, Royal College of Surgeons in Ireland, Dublin, Ireland; ^7^ UCD School of Biomolecular and Biomedical Science, UCD Conway Institute, University College Dublin, Dublin, Ireland; ^8^ Department of Molecular and Cellular Therapeutics, Royal College of Surgeons in Ireland, Dublin, Ireland; ^9^ Department of Medicine II, University Hospital Mannheim, Medical Faculty Mannheim, Heidelberg University, Mannheim, Germany; ^10^ Institute of Pathology, University Hospital Mannheim, Medical Faculty Mannheim, Heidelberg University, Mannheim, Germany

**Keywords:** apelin, biomarker, bevacizumab response, VEGF, colorectal cancer

## Abstract

Bevacizumab (bvz) is currently employed as an anti-angiogenic therapy across several cancer indications. Bvz response heterogeneity has been well documented, with only 10-15% of colorectal cancer (CRC) patients benefitting in general. For other patients, clinical efficacy is limited and side effects are significant. This reinforces the need for a robust predictive biomarker of response. To identify such a biomarker, we performed a DNA microarray-based transcriptional profiling screen with primary endothelial cells (ECs) isolated from normal and tumour colon tissues. Thirteen separate populations of tumour-associated ECs and 10 of normal ECs were isolated using fluorescence-activated cell sorting. We hypothesised that VEGF-induced genes were overexpressed in tumour ECs; these genes could relate to bvz response and serve as potential predictive biomarkers. Transcriptional profiling revealed a total of 2,610 differentially expressed genes when tumour and normal ECs were compared. To explore their relation to bvz response, the mRNA expression levels of top-ranked genes were examined using quantitative PCR in 30 independent tumour tissues from CRC patients that received bvz in the adjuvant setting. These analyses revealed that the expression of *MMP12* and *APLN* mRNA was significantly higher in bvz non-responders compared to responders. At the protein level, high APLN expression was correlated with poor progression-free survival in bvz-treated patients. Thus, high APLN expression may represent a novel predictive biomarker for bvz unresponsiveness.

## INTRODUCTION

Current clinically approved angiogenesis inhibitors primarily target the vascular endothelial growth factor (VEGF) signalling pathway, thereby inhibiting tumour vascularisation [1]. VEGF is produced and secreted by tumour cells and binds to the VEGF receptor (VEGFR/KDR), which is primarily expressed on non-tumourigenic endothelial cells (ECs) that line the inner side of blood vessels. Activation of the VEGF signalling pathway triggers sprouting and extension of new blood vessels from pre-existing blood vessels into the growing tumour. These new vessels provide the tumour with oxygen and nutrients enabling the tumour to grow beyond 1-2 mm [2]. VEGF signalling is one of the most ubiquitous growth factor signalling pathways to regulate angiogenesis under normal physiological, but also pathological conditions [3]. Although the inhibition of angiogenesis seems to be an effective strategy, the responsiveness of cancer patients to currently approved angiogenesis inhibitors is limited with significant drug-related side effects and rapid acquired drug resistance [4–9]. For example, it has been shown that blockade of the VEGF signalling by anti-angiogenic factors results in compensation by other pro-angiogenic factors and signalling pathways, which can explain the limited efficacy and rapid development of resistance for current anti-VEGF/VEGFR therapies [1, 10, 11].

Bevacizumab (bvz) is an anti-angiogenic monoclonal antibody that binds VEGF, thereby inhibiting the activation of the VEGF signalling pathway [12]. As a single agent, it is approved (in the United States) for glioblastoma patients that do not respond to standard-of-care therapy. In terms of combination therapy, bvz is currently used for metastatic colorectal cancer (CRC), non-small cell lung cancer, ovarian cancer, cervical cancer, and metastatic renal cell cancer. Previously, bvz has been used for treatment of metastatic breast cancer. However, FDA approval was rescinded after several studies could not prove a significant and enduring clinical benefit [4, 8, 9]. The response rates of bvz are relatively modest in most cancer types. Indeed, only 10-15% of metastatic CRC patients benefit from combined bvz/chemotherapy treatment [13–16] and only a 13% increased response has been observed for non-small lung cancer patients when treated with combined bvz/chemotherapy compared to chemotherapy alone [6]. Higher response rates have been shown in glioblastoma patients receiving bvz mono- or combination therapy; bvz response rate was 29-42% [5, 7]. Major side effects associated with bvz use are cardiovascular problems, bleeding, renal toxicity, and rare gastrointestinal perforations [17, 18].

The overall modest response rate and significant adverse events associated with bvz use emphasise the need for a *bona fide* biomarker that predicts responsiveness, thereby preventing bvz overtreatment and morbidity in patients that do not benefit from this therapy. Currently, a validated predictive biomarker has not been identified. Since bvz targets VEGF signalling, we hypothesised that differential expression of genes regulating this pathway could have clinical utility as potential predictive biomarkers, as they could interfere with bvz efficacy. Moreover, the expression levels of these factors prior to commencement of bvz therapy could be indicative for bvz response.

As VEGF signalling is more prominent in hypoxic tumour-associated ECs (TECs) compared to quiescent normal ECs (NECs) [3], genes that are indirectly or directly regulated by VEGF signalling may be identified by comparing the expression profile of TECs and NECs. To this end, a selective transcriptomic screen was performed on TECs and NECs isolated from CRC patients. An expression profile comparison of these two cell types revealed a unique gene signature for TEC-specific genes. The mRNA expression levels of top-ranked genes from this signature were independently verified in a separate cohort of primary resected tissues from bvz-responding and non-responding CRC patients.

We identified *APLN* and *MMP12* mRNA expression levels as candidate predictive markers for bvz therapy. The potential role of *APLN* as a predictive marker was further confirmed by immunohistochemistry; high APLN protein levels were detected in bvz non-responders and associated with poor progression-free survival rates in these patients.

## RESULTS

### Tumour-associated endothelial cell signature in primary CRC

In order to identify genes specifically expressed in endothelial cells (ECs), single cell sorting was performed on 10 normal colon and 13 tumour adenocarcinoma (CRC) patient tissues (non-paired) using flow cytometry. For this, the EC marker PECAM1 (CD31) was used in combination with the CD45 leukocyte marker (Supplementary Figure 1), to exclude CD31^+^ leukocytes (e.g. monocytes). The absolute number of normal or tumour ECs (CD31^+^CD45^−^) within the whole tissue population differed substantially between patients, but only represented ~1% of cells on average (data not shown). ECs were enriched up to 75-95% purity. Transcriptomic profiling using DNA microarray technology and subsequent Limma analysis comparing TEC samples and NEC samples revealed a TEC-specific signature that included many EC-specific genes such as *VWF* (fold change (FC) 2.4, false discovery rate (FDR) *p*=0.03), *MCAM* (FC 1.93, FDR *p*=0.006), *ESM1* (FC 1.51, FDR *p*=0.006), *NOTCH4* (FC 1.43, FDR *p*=0.01), *CD34* (FC 1.37, FDR *p*=0.04) [19–22] and several genes encoding for extracellular membrane-associated proteins like collagens. This reflects the active state of TECs in contrast to NECs. Furthermore, other previously reported TEC-specific markers were also identified, like *CLEC14A* (FC 1.47, FDR *p*=0.05), *PCDH17* (FC 2.43, FDR *p*=0.0007), *ROBO4* (FC 1.40, FDR *p*=0.03) and *BIRC5* (FC 1.19, FDR *p*=0.03) [23–27]. In order to explore endothelial-specificity of significantly expressed genes, we also compared the gene expression profile of whole CRC tumours (*n*=26) with that of normal colon tissue (*n*=5) to define global gene expression differences of tumour cells and its surrounding microenvironment (i.e. tumour tissue signature). Comparing TEC-specific genes (P<0.05, excluding probe sets without gene annotation) to the tumour signature revealed that only 375 (18%) of the TEC-specific genes were also present in the whole tumour signature and may therefore also be highly expressed by non-endothelial cells. As over 80% of TEC-specific genes were not part of the whole tumour signature, this shows that isolating and profiling an under-represented single cell population, such as the EC compartment, reveals a highly selective set of tumour vasculature-specific genes. Using this approach, a unique set of CRC vasculature-specific genes has been identified.

### Enrichment of VEGF signalling genes in the TEC-specific signature

**Figure 1:**
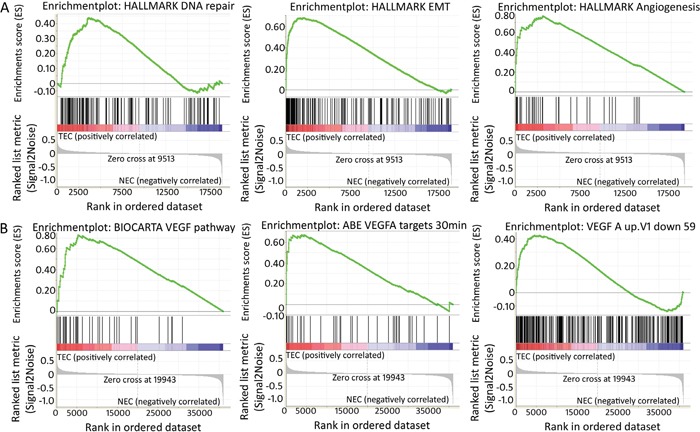
Enriched gene sets within TEC signature (GSEA) **(A)** Enrichment plots generated by the GSEA tool of the three most enriched gene sets of the MSigDb “hallmark gene set”. These are *a priori* defined gene sets that represent specific well-defined biological states or processes, i.e. from left to right, DNA repair, epithelial-mesenchymal transition and angiogenesis. In each analysis, genes within pre-defined gene sets are ranked and scored based on the position within the TEC-specific signature. In each plot, “1” corresponds to NEC and “2” to TEC. Each vertical line of the barcode represents a gene. Most genes on the left positively correlate with TEC samples, most genes on the right correlate negatively with TEC samples. Lines in between represent genes that are not differentially expressed between TECs and NECs. As vertical lines of the barcode are overrepresented at the left side of the graph, this means many of the genes within the pre-defined gene set are positively correlated/enriched within TECs. The score relating to this enrichment (enrichment score or ES) is indicated by the green line. **(B)** GSEA enrichment plots show enrichment of genes involved in VEGF signalling in TEC samples. The first plot corresponds to genes assigned to the VEGF pathway by the Biocarta database, the second and third plot correspond to published data of genes up-or downregulated after treatment of HUVEC cells with VEGFA (Pubmed 12197474 respectively Pubmed 15516835) [64, 65].

Gene set enrichment analysis (GSEA) was performed to identify specific gene expression profiles that were enriched in TEC samples compared to NEC samples. For this, all probes sets were loaded and technical sample replicates were discarded to minimise statistical noise, which reduced the sample size to 9 TEC and 8 NEC samples. Based on these data, GSEA generated a TEC vs NEC profile (GSEA TEC profile) and compared this to all Molecular Signatures Database (MSigDB) “hallmark gene sets”, which represent specific well-defined biological states or processes (*n*=50). The GSEA TEC profile was enriched for 10 of these gene sets (FDR <0.25, Supplementary Table 2) that were associated with a normalised enrichment score (NES) ranging from 1.36-1.49. The three most significantly enriched gene sets comprised angiogenesis, DNA repair, and epithelial-to-mesenchymal transition (EMT) (Figure 1A, FDR 0.15, 0.13, and 0.14, respectively). Identifying angiogenesis as one of the top active processes confirmed the highly selective EC isolation approach used in this study.

As bvz blocks VEGF signalling, it was hypothesised that a potential bvz response-predictive biomarker may be linked or regulated by VEGF signalling. Therefore, GSEA was performed to investigate the presence of VEGF-induced genes. Accordingly, the GSEA TEC profile was compared to VEGF-oriented signatures (see Material and Methods) provided by MSigDB (v5.1). GSEA showed enrichment of three VEGF signalling pathway gene sets (FDR <0.25, Figure 1B). This enrichment confirmed that the GSEA TEC profile includes VEGF signalling genes, which may reflect genes that potentially affect bvz response. This was further confirmed by comparison of the GSEA TEC profile with the signature of Jones and co-workers [27] who published a list of differentially expressed genes following bvz treatment in highly vascularized glioblastoma U87 xenografts. Comparison of our GSEA TEC profile showed strong enrichment for the genes identified in this study (ES 0.5, Nom *p*-value 0.02).

### High *APLN* and *MMP12* mRNA levels in bvz non-responding patient samples

**Figure 2:**
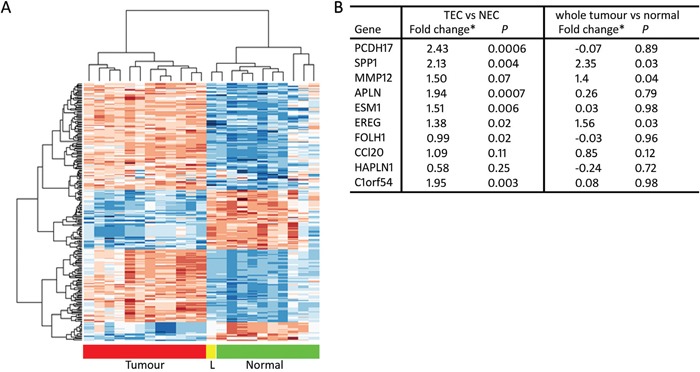
TEC-specific signature **(A)** Heatmap showing hierarchical clustering of the 200 most significantly differentially expressed probe sets between TEC and NEC. Samples are depicted on the horizontal axis, genes on the vertical axis. “L” means liver metastasis. Within the heatmap, red is highly expressed and blue is lowly expressed. **(B)** Table representing microarray Limma fold changes and FDR-corrected *p*-value of TEC-specific candidate genes comparing TEC with NEC samples, as well as Limma fold changes and FDR-corrected *p*-value comparing whole tumour and normal tissues.

The TEC-specific signature that we generated was represented by 2,610 unique probe sets, including probe sets without gene annotation (Figure 2A, top200 ranked on p-value). The top 500 with the highest positive fold change (FC) were extracted for further validation. These corresponded to 244 single genes and 55 probe sets without annotation. Genes were further selected via a candidate approach, based on their biological function and cell expression distribution. This resulted in a bvz-predictive candidate list of 10 genes: *PCDH17*, *SPP1*, *MMP12*, *APLN*, *ESM1*, *EREG*, *FOLH1*, *CCL20*, *HAPLN1* and *C1orf54* (Figure 2B). Only *SPP1* and *MMP12* were also present in the whole tumour signature, suggesting that these targets are also abundantly expressed in other cell types, in addition to ECs.

**Figure 3:**
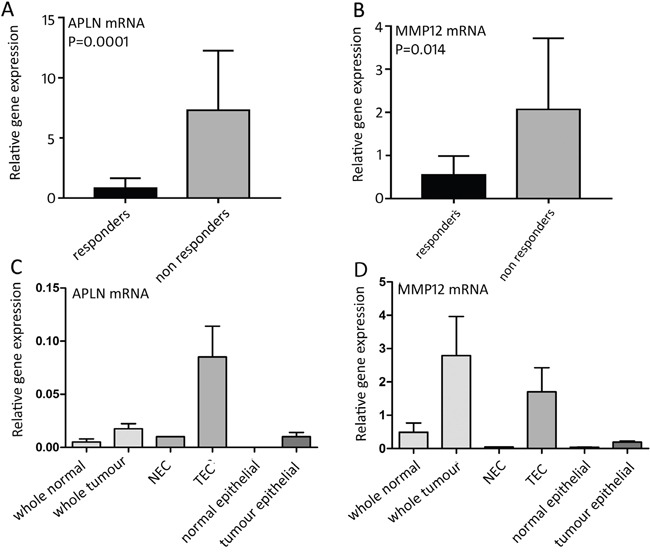
mRNA expression of bvz response-predicting genes **(A)** Relative *APLN* (*p*=0.0001) and **(B)** *MMP12* (*p*=0.0140) mRNA expression between 15 bvz-responding and 15 non-responding patients. Data is represented as mean ± SD. **(C)** and **(D)** *APLN* and *MMP12* mRNA expression, respectively, in different cell fractions: normal tissue (*n*=4), tumour tissue (*n*=4), NEC (*n*=1), TEC (APLN *n*=4, MMP12 *n*=3), isolated normal epithelial cells (*n*=4) and tumour epithelial cell samples (*n*=4). Data is represented as mean ± SD.

The mRNA expression levels of these candidate genes were examined by qRT-PCR analysis in an independent set of CRC patient tissue samples that were collected prior to bvz treatment (15 responders versus 15 non-responders). This revealed a significant difference in *APLN* and *MMP12* mRNA expression levels between responding and non-responding patients (Figure 3A and 3B). *APLN* and *MMP12* mRNA levels were low in bvz-responding patients and high in non-responding CRC patients (*p*=0.0001 and *p*=0.0140, respectively). No difference in expression was observed for all other eight candidates (data not shown).

Based on this, only *APLN* and *MMP12* were further assessed through qRT-PCR analysis using sorted cell samples that were isolated parallel to the initial sorted patient set used for microarray analysis. For this, we used non-paired samples for which enough material was available. This corresponded to normal tissue (*n*=4), tumour tissue (*n*=4), NEC (*n*=1), TEC (APLN *n*=4, MMP12 *n*=3), isolated normal epithelial cells (*n*=4) and tumour epithelial cell samples (*n*=4), which were isolated following the same approach (Figure 3C and 3D). Epithelial samples were selected based on EpCAM expression. *APLN* expression was barely detectable in both normal and tumour whole tissue samples, as well as in normal and tumour epithelial cells and NECs, although a slight elevation was observed in whole tumour samples when compared to their normal counterparts. The contradiction of this slight elevation compared to the microarray data, in which *APLN* was equally expressed in whole tissue samples of tumour and bulk (Figure 3B), can be explained by the fact that the qRT-PCR approach is more sensitive. As expected, *APLN* levels were high in isolated TECs. *MMP12* mRNA expression was barely detectable in normal tissue samples and showed elevated expression in TECs. *MMP12* was also clearly expressed (although at low levels) in whole tumour samples confirming that *MMP12* is also expressed in other cell types in addition to ECs, as previously discussed.

### High APLN protein expression in bvz non-responding patients

To confirm the association between *APLN* or *MMP12* mRNA expression and bvz response at the protein level, immunohistochemistry (IHC) was performed. For this, we used the same samples (described above) from the independent set of whole tumour CRC patient tissues. Initially, fifteen responder and 15 non-responder tissue sections, along with corresponding healthy control tissue sections, were immunostained using commercial antibodies against both targets. Due to poor tissue quality (e.g. tissue folding or lack of tumour tissue), several APLN-stained samples were excluded, which reduced sample sizes for the analysis to 10 responders versus 10 non-responders. Sections were scanned using a 3D Histech Pannoramic 250 Flash scanner and image analysis was performed using IHC-MARK.

**Figure 4:**
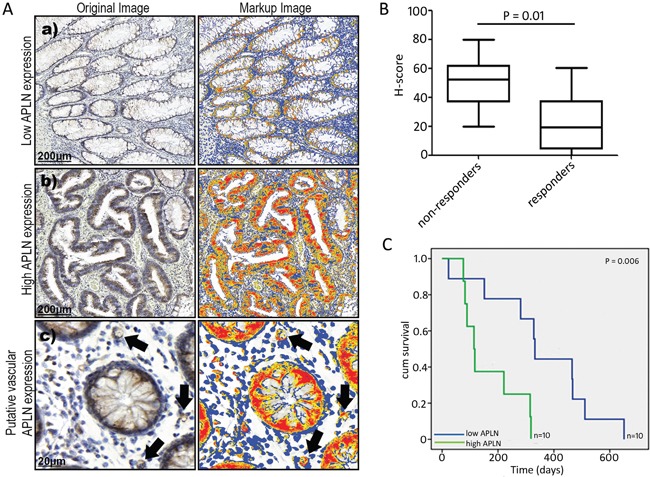
APLN protein expression in CRC tissues **(A)** (a and b) represent images of low and high APLN protein expression in tumour tissue sections, as determined with immunohistochemistry. Subfigure (c) illustrates vascular APLN expression, denoted by arrows. **(B)** Box plot (median, 25th and 75th quartiles) demonstrating the distribution of quantitative APLN data (H-score) obtained from the automated image analysis data of 10 bvz responders and 10 non-responders. *P* value corresponds to difference in median value of H-scores between the non-responders and responders. **(C)** Kaplan–Meier survival plot showing progression-free survival. Patients are categorised based on APLN expression. Y-axis shows cumulative (cum) survival, X-axis shows time (days). Patients with low APLN levels (*n*=10) are indicated in blue, patients with high APLN levels (*n*=10) are indicated in green. Log-rank *p*-value is shown.

Surprisingly, no difference in MMP12 protein expression was detected between bvz responders versus non-responders (data not shown). On the other hand, image analysis data (H-Score) showed a significantly (*p*=0.01) higher APLN protein expression in tumour tissues from the non-responder patient group compared to the responder group (Figure 4A and 4B). Surprisingly, a patient-by-patient analysis revealed low correlation between APLN mRNA and protein expression (Pearson's r=0.18 and 0.04 for responders and non-responders, respectively). High APLN protein expression in tumour tissues was significantly associated with decreased progression-free survival (PFS) in bvz-treated patients (high APLN = 3.8 months, low APLN = 11.06 months, *p*=0.006, Figure 4C).

## DISCUSSION

In recent years, several research groups have attempted to identify a biomarker for bvz response. Hypertension, a bvz-associated adverse event, was suggested to be predictive for bvz response, as well as genomic markers (e.g. SNPs in *VEGF* receptors) or a set of multiple VEGF signalling genes [2, 28–32]. In addition, neuropilin-1 and circulating small isoforms of VEGFA were recently identified in advanced or metastatic gastric cancer as potentially strong predictive markers [31]. Also, circulating VEGFR2/KDR levels have recently been proposed as a candidate biomarker for bvz in metastatic CRC patients [32]. However, these studies still need additional validation. Moreover, although one might expect that VEGF itself may have a *bona fide* predictive value, no studies have shown a robust correlation [2, 33, 34].

In this study, we identified APLN as a potential bvz response-predictive biomarker in CRC patients. We observed that bvz non-responding patients exhibit high levels of APLN. In contrast to other putative bvz biomarkers, which are not directly linked to the tumour vascularisation or are related to various functions in tumour cells, APLN is produced and secreted by TECs directly. Previously, several research groups evaluated APLN differences between normal and tumour transcriptomic profiles [24, 26, 27, 35–41]. A study by Picault and co-workers showed high APLN levels in 50% of CRC samples analysed [42]. Overexpression of APLN and its receptor APJ is further detected in highly proliferating micro-vessels in primary glioblastoma tissues when compared to normal brain tissues [43], providing further evidence for the vital role of APLN during tumour angiogenesis. We are the first who provide evidence for an association between differential APLN expression and bvz response.

A possible explanation for non-responsiveness to bvz in patients with high APLN levels could be the VEGF-like function of APLN, which may be able to compensate for loss of VEGF signalling [44, 45]. APLN functions in an autocrine and paracrine manner and binds to the APJ G-protein-coupled receptor, thereby inducing inhibition of adenylyl cyclase and phosphorylation of ERK, AKT and p70 S6 kinase. Expression of the APLN receptor has been identified on colon epithelial cells of clinical samples by IHC and, thereafter, functionality of this signalling route was shown in colon cell line models with various APLN fragments [46, 47]. One of the factors that drives *APLN* gene expression is hypoxia [48, 49] and a HIF1α-responsive element has been identified in the *APLN* gene. Furthermore, it has been previously demonstrated that APLN promotes embryonic and tumour angiogenesis, as well as tumour growth *in vivo* [48]. Similar to VEGF, APLN has been shown to act as vascular chemo-attractant [43] and its expression is induced in sprouting vessels under pathological conditions e.g. in tumours [50–52]. Recently, it has been shown that administration of recombinant APLN protein partially restores the VEGF/FGF2-induced angiogenic response in the retina of APLN knockout mice [53]. These studies demonstrate a prominent role for APLN in promoting neovascularisation. All this suggests that tumour endothelial-derived APLN expression may compensate for bvz-induced loss of VEGF signalling, by fulfilling a comparable pro-angiogenic function. These data further support our hypothesis that high levels of APLN can compensate for loss of VEGF signalling caused by bvz, thereby inducing bvz resistance.

Interestingly, we further noticed that high APLN levels were associated with poor PFS in CRC patients. A similar association of APLN with poor outcome has also been noticed for other cancer indications [54, 55]. However, we do not know the role for APLN in bvz-untreated CRC patients and consequently cannot exclude a prognostic role for APLN expression. In addition, APLN has previously been associated with tumour angiogenesis and survival in other cancer indications. High APLN levels in non-small cell lung cancer patients were correlated with high micro-vessel density [55] and high APLN serum levels were also correlated with disease progression in a study that examined patients that suffered from either lung, gastrointestinal, breast, prostate or gynaecologic cancer [56]. These levels may reflect the state of vascularisation in these tumours and further underlines the importance of APLN during tumour vascularisation.

Recently, Zhang and co-workers described a decrease in APLN levels following bvz treatment in mice, resulting in stabilisation of the tumour vasculature [57]. Interestingly, they also described some preliminary data from an ongoing clinical trial, in which low APLN levels were observed in serum of cancer patients that respond well to bvz treatment. These data further support our findings regarding APLN as a potential biomarker for bvz responsiveness.

One of the limitations of our study is the relatively small cohort of CRC patients assessed. Therefore, the predictive value of this putative marker requires confirmation in a larger cohort of patients. A second limiting factor is that we do not know the distribution of APLN expression throughout the whole tumour. Therefore, our results could be biased due to the position at which the biopsy of the tumour had been extracted. To circumvent issues relating to intra-tumoural variation, APLN levels could be assessed in serum. This approach may be relevant for clinical diagnostics. However, APLN serum concentration is correlated to body mass and has been shown to contribute to changes in adiposity observed in overweight and obese subjects [58]. This could complicate the use of serum APLN as a diagnostic marker for bvz responsiveness.

In summary, we have identified APLN as putative predictive bvz biomarker in CRC patients. High APLN levels predict a poor response to bvz therapy in CRC. Although our results need confirmation in a larger patient cohort, this study shows for the first time that APLN expression may be used to predict bvz responsiveness in CRC patients and potentially also for other cancer types.

## MATERIALS AND METHODS

### Patient cohort

Fresh normal and tumour colon tissues for single-cell isolation were obtained after surgery from the VU Medical Center (VUmc) and the ‘Onze Lieve Vrouwe Gasthuis’ (OLVG). Tumour tissue samples that were used for verification were collected at University Hospital Mannheim, Heidelberg University, Mannheim, Germany. For this, thirty patients undergoing palliative chemotherapy in combination with bvz were randomly selected from a larger cohort of CRC patients receiving bvz treatment [59]. Resected tumours and tumour-associated normal tissues were preserved as formalin-fixed paraffin embedded (FFPE) tissue for full-face sectioning. Response to bvz therapy was defined as 10 months progression-free survival while the patient was on bvz treatment. Clinicopathological details, including gender, age at start of bvz treatment, T- and N-classification of the primary tumour, tumour grade, and chemotherapy backbone administered together with bvz, are depicted in Table 1. The study had full ethical approval from local ethics committees involved in the study, with patients giving informed consent for use of tissue for research according to the Declaration of Helsinki.

**Table 1:** Clinicopathological characteristics

	Responders*	Non-responders
**Gender**		
male	9	8
female	6	7
**Median age**	63	66
**T-classification**		
1	0	0
2	4	1
3	8	10
4	3	3
**N-classification**		
0	6	5
1-2	8	9
**Localisation of primary**		
colon	8	10
rectum	7	5
**Chemotherapy (+ bvz)**		
oxaliplatin doublet	2	5
irinotecan doublet	8	7
fluoropyrimidine + mitomycin	0	2
fluoropyrimidine	5	1

*Responder patient group was categorised based on response to bvz therapy as determined by 10 months progression-free survival.

### Tissue processing and cell sorting

Fresh tissues (>0.5 g) for single-cell isolation were obtained directly after surgery and stored in 10% FCS at 4°C for a maximum of 16 hr. Normal adjacent tissues were obtained >10 cm from the bulk tumour but processing often failed due to the rigidity of normal tissue samples. Excess blood was removed by a PBS wash. Tissues were dispersed into ~4 mm pieces using razor blades. Further dissociation of cells was enzymatically obtained using collagenase I (0.2%) and dispase (2.5 U/mL) in DMEM with 2% FCS, accompanied by actinomycin D (74 μg/mL), DNAse (25 KU), and calcium chloride dehydrate (5 mM). The solution was incubated in a T25 filter flask for 1 hr at 37°C while shaking. After incubation, cells were sieved through a 100 μm nylon filter and spun down. Red blood cells were lysed in shock medium for 10 min on ice. After two washes, cells were stained for 30 min with anti-PECAM1-PE (clone WM59, ABD Serotec, Kidlington, UK), anti-CD45-APC (clone 2D1, BD, San Jose, CA, USA) and anti-EpCAM-FITC (clone 9c4, Biolegend, San Diego, CA, USA). Cells were washed, filtered (100 μm) and subjected to fluorescence-activated cell sorting (FACS Aria, BD) using a 100 μm nozzle (25 psi). Various cell populations are indicated in Supplementary Figure 1. Collected cells were spun down and immediately dissolved into 500 μL TRIzol (ThermoFisher Scientific, Waltham, MA, USA).

### DNA microarray profiling

RNA extraction and microarray hybridisation procedures were essentially performed according to Martens-de Kemp [60]. Total RNA was isolated from (sorted) cells in 0.5 mL of TRIzol. RNA integrity and concentrations were measured with a Bioanalyzer 2100 (Agilent Technologies, Santa Clara, CA, USA) and Nanodrop spectrophotometer ND-1000 (ThermoFisher Scientific). Fifty nanograms total RNA per sample was used as an input for amplification and labelling with the “Low Input Quick Amp Labelling kit” (Agilent Technologies). Labelled RNA was purified using the RNeasy Mini Kit (Qiagen Ltd., Venlo, the Netherlands). Labelled samples were hybridised onto whole human genome GE 4×44K microarrays (G4112F, Agilent Technologies) according to the manufacturer's protocol (Agilent Technologies). Samples were hybridised in three different batches and each batch contained technical replicates on each slide. Scanning was performed using a microarray scanner G2505B (Agilent Technologies) and Feature Extraction v10.7.3.1 using the manufacturer's protocols (Agilent Technologies). The microarray data have been submitted to the National Center for Biotechnology Information (NCBI) Gene Expression Omnibus database (GEO, database number: GSE89287) (http://www.ncbi.nlm.nih.gov/geo).

### qRT-PCR

From parallel-sorted samples and from the tumour cohort that was used for verification, RNA was isolated using TRIzol reagent (ThermoFisher Scientific). Complementary DNA (cDNA) was generated from 1 μg RNA per sample. cDNA and RNA were stored at -80°C. Primers to measure transcripts in sorted cell fractions were *APLN* Fw 5′ CTCTGGCTCTCCTTGACCG 3′, Rv 5′ GGCCCATTCCTTGACCCTC 3′ and *MMP12* Fw 5′ CAAAGGCCGTAATGTTCCCC 3′, Rv 5′ GGGTCTCCATACAGGGACTGA 3′. Levels of candidate transcripts in independent whole tumour tissues were determined using Array Microfluidic Cards with TaqMan fluorogenic gene expression probe sets (ABI Biosystems, CA, USA).

### Immunohistochemistry

For APLN IHC, we used a polyclonal rabbit antibody targeted against the C-terminal region of the human protein (ab59469, Abcam, Cambridge, UK). For MMP12, a rabbit monoclonal antibody was used (clone EP1261Y, ab52897, Abcam). The negative control reagent was a polyclonal rabbit IgG isotype control (ab27472, Abcam). Deparaffinisation, rehydration, and target retrieval was performed in a PT Link system (Dako, Glostrup, Denmark). Slides were then processed on the Autostainer Link 48 (Dako) using an automated staining protocol validated for the APLN and MMP12 assay. The IHC staining protocol included sequential application of a peroxidase-blocking reagent, primary antibody (anti-APLN or anti-MMP12) or negative control reagent, mouse anti-rabbit IgG linker, visualisation reagent consisting of secondary antibody molecules and horseradish peroxidase coupled to a polymer backbone, 3,30-diaminobenzidine tetrahydrochloride (DAB) chromogen reagent with hydrogen peroxide substrate, and a DAB enhancer which modifies the colour of the precipitated chromogen. Automated staining runs included a control slide containing a Hela cell pellet as a positive control and a ‘no primary antibody’ on a Hela cell pellet as a negative control. Reagents utilised in addition to the APLN and MMP12 assays included a wash buffer specially formulated for automated IHC staining, and a haematoxylin counterstain. IHC-stained slides were mounted in non-aqueous, permanent mounting media.

### Image analysis

Glass slides stained via IHC were converted to digital format using 3D Histech Pannoramic 250 Flash glass slide scanner. The images were scanned at 40x magnification with 0.20 μm resolution using brightfield mode. Using Pannaromic whole slide image viewer, both tumour and non-tumour regions were manually annotated, extracted and stored as tiff-format to perform image analysis. Using an in-house image analysis software IHC-MARK [61], the stained images were separated into DAB and haematoxylin channels using a colour-deconvolution algorithm and their relative protein expression was quantified only from the DAB channel for both tumour and non-tumour regions. The deconvolution parameters were optimally selected by considering different samples at multiple regions. H-score was calculated for each image and scores were used for the generation of survival curves.

### Statistical analysis

Gene expression data was LOESS-normalised and were, after quality control, further normalised according to the variance stabilisation (VSN) method to correct for intra- and inter-array signal variation, respectively [62]. VSN and Linear models for microarray data (Limma) analyses were performed using Bioconductor software release 3.1.2. TECs were identified using Limma analysis to reveal differentially expressed genes between TECs and NECs [63]. This analysis was corrected for batch effect and technical replicates. *P*-values were adjusted for multiple testing using the Benjamini and Hochberg False Discovery Rate (FDR). Positive fold change (FC) meant higher expression in TEC versus NEC, and vice versa.

Gene set enrichment analysis (GSEA) v2.2.2 was performed to identify previously reported Molecular Signatures Database (MSigdb, v5.1) gene signatures or pathways within our TEC signature. One thousand permutations of the phenotype were used and our signature was compared with the various gene sets provided in Supplementary Table 1 [64, 65]. Normalised enrichment score (NES) and FDR were used when 3-10 gene sets were compared, whereas enrichment score (ES) and nominal *p*-value (nom p-value) were used when only 1 gene set was tested. FDR values ≤ 0.25 (25%) and nom *p*-values ≤ 0.05 were regarded as significant. Mann-Whitney U statistics were used to compare the significance in mRNA expression levels (as determined by qRT-PCR) between bvz/chemotherapy responders and non-responders. Kaplan-Meier survival curves of progression-free survival were generated based on APLN protein expression: strong (high) or weak (low) staining. To determine this, median H-score values were used. Kaplan–Meier survival plots and log-rank *P*-values for individual proteins were calculated using SPSS Statistics 18.0 (IBM SPSS Statistics, Somers, NY, USA), with *p*-values ≤ 0.05 considered to be significant.

## SUPPLEMENTARY FIGURE AND TABLES



## References

[1] Bergers G, Benjamin LE (2003). Tumorigenesis and the angiogenic switch. Nat Rev Cancer.

[2] Jubb AM, Hurwitz HI, Bai W, Holmgren EB, Tobin P, Guerrero AS, Kabbinavar F, Holden SN, Novotny WF, Frantz GD, Hillan KJ, Koeppen H (2006). Impact of vascular endothelial growth factor-A expression, thrombospondin-2 expression, and microvessel density on the treatment effect of bevacizumab in metastatic colorectal cancer. J Clin Oncol.

[3] Olsson AK, Dimberg A, Kreuger J, Claesson-Welsh L (2006). VEGF receptor signalling - in control of vascular function. Nat Rev Mol Cell Biol.

[4] Brufsky AM, Hurvitz S, Perez E, Swamy R, Valero V, O’Neill V, Rugo HS (2011). RIBBON-2: a randomized, double-blind, placebo-controlled, phase III trial evaluating the efficacy and safety of bevacizumab in combination with chemotherapy for second-line treatment of human epidermal growth factor receptor 2-negative metastatic breast cancer. J Clin Oncol.

[5] Friedman HS, Prados MD, Wen PY, Mikkelsen T, Schiff D, Abrey LE, Yung WK, Paleologos N, Nicholas MK, Jensen R, Vredenburgh J, Huang J, Zheng M (2009). Bevacizumab alone and in combination with irinotecan in recurrent glioblastoma. J Clin Oncol.

[6] Johnson DH, Fehrenbacher L, Novotny WF, Herbst RS, Nemunaitis JJ, Jablons DM, Langer CJ, DeVore RF, Gaudreault J, Damico LA, Holmgren E, Kabbinavar F (2004). Randomized phase II trial comparing bevacizumab plus carboplatin and paclitaxel with carboplatin and paclitaxel alone in previously untreated locally advanced or metastatic non-small-cell lung cancer. J Clin Oncol.

[7] Kreisl TN, Kim L, Moore K, Duic P, Royce C, Stroud I, Garren N, Mackey M, Butman JA, Camphausen K, Park J, Albert PS, Fine HA (2009). Phase II trial of single-agent bevacizumab followed by bevacizumab plus irinotecan at tumor progression in recurrent glioblastoma. J Clin Oncol.

[8] Miles DW, Chan A, Dirix LY, Cortes J, Pivot X, Tomczak P, Delozier T, Sohn JH, Provencher L, Puglisi F, Harbeck N, Steger GG, Schneeweiss A (2010). Phase III study of bevacizumab plus docetaxel compared with placebo plus docetaxel for the first-line treatment of human epidermal growth factor receptor 2-negative metastatic breast cancer. J Clin Oncol.

[9] Robert NJ, Dieras V, Glaspy J, Brufsky AM, Bondarenko I, Lipatov ON, Perez EA, Yardley DA, Chan SY, Zhou X, Phan SC, O’Shaughnessy J (2011). RIBBON-1: randomized, double-blind, placebo-controlled, phase III trial of chemotherapy with or without bevacizumab for first-line treatment of human epidermal growth factor receptor 2-negative, locally recurrent or metastatic breast cancer. J Clin Oncol.

[10] Huijbers EJ, van Beijnum JR, Thijssen VL, Sabrkhany S, Nowak-Sliwinska P, Griffioen AW (2016). Role of the tumor stroma in resistance to anti-angiogenic therapy. Drug Resist Updat.

[11] van Beijnum JR, Nowak-Sliwinska P, Huijbers EJ, Thijssen VL, Griffioen AW (2015). The great escape; the hallmarks of resistance to antiangiogenic therapy. Pharmacol Rev.

[12] Ferrara N, Gerber HP, LeCouter J (2003). The biology of VEGF and its receptors. Nat Med.

[13] Hurwitz H, Fehrenbacher L, Novotny W, Cartwright T, Hainsworth J, Heim W, Berlin J, Baron A, Griffing S, Holmgren E, Ferrara N, Fyfe G, Rogers B (2004). Bevacizumab plus irinotecan, fluorouracil, and leucovorin for metastatic colorectal cancer. N Engl J Med.

[14] Giantonio BJ, Catalano PJ, Meropol NJ, O’Dwyer PJ, Mitchell EP, Alberts SR, Schwartz MA, Benson AB, Eastern Cooperative Oncology Group Study E (2007). Bevacizumab in combination with oxaliplatin, fluorouracil, and leucovorin (FOLFOX4) for previously treated metastatic colorectal cancer: results from the Eastern Cooperative Oncology Group Study E3200. J Clin Oncol.

[15] Hochster HS, Hart LL, Ramanathan RK, Childs BH, Hainsworth JD, Cohn AL, Wong L, Fehrenbacher L, Abubakr Y, Saif MW, Schwartzberg L, Hedrick E (2008). Safety and efficacy of oxaliplatin and fluoropyrimidine regimens with or without bevacizumab as first-line treatment of metastatic colorectal cancer: results of the TREE Study. J Clin Oncol.

[16] Price TJ, Zannino D, Wilson K, Simes RJ, Cassidy J, Van Hazel GA, Robinson BA, Broad A, Ganju V, Ackland SP, Tebbutt NC (2012). Bevacizumab is equally effective and no more toxic in elderly patients with advanced colorectal cancer: a subgroup analysis from the AGITG MAX trial: an international randomised controlled trial of Capecitabine, Bevacizumab and Mitomycin C. Ann Oncol.

[17] Verheul HM, Pinedo HM (2007). Possible molecular mechanisms involved in the toxicity of angiogenesis inhibition. Nat Rev Cancer.

[18] Chen HX, Cleck JN (2009). Adverse effects of anticancer agents that target the VEGF pathway. Nat Rev Clin Oncol.

[19] Ucuzian AA, Gassman AA, East AT, Greisler HP (2010). Molecular mediators of angiogenesis. J Burn Care Res.

[20] Fina L, Molgaard HV, Robertson D, Bradley NJ, Monaghan P, Delia D, Sutherland DR, Baker MA, Greaves MF (1990). Expression of the CD34 gene in vascular endothelial cells. Blood.

[21] Shih IM (1999). The role of CD146 (Mel-CAM) in biology and pathology. J Pathol.

[22] Sarrazin S, Adam E, Lyon M, Depontieu F, Motte V, Landolfi C, Lortat-Jacob H, Bechard D, Lassalle P, Delehedde M (2006). Endocan or endothelial cell specific molecule-1 (ESM-1): a potential novel endothelial cell marker and a new target for cancer therapy. Biochim Biophys Acta.

[23] Mura M, Swain RK, Zhuang X, Vorschmitt H, Reynolds G, Durant S, Beesley JF, Herbert JM, Sheldon H, Andre M, Sanderson S, Glen K, Luu NT (2012). Identification and angiogenic role of the novel tumor endothelial marker CLEC14A. Oncogene.

[24] Zhuang X, Herbert JM, Lodhia P, Bradford J, Turner AM, Newby PM, Thickett D, Naidu U, Blakey D, Barry S, Cross DA, Bicknell R (2015). Identification of novel vascular targets in lung cancer. Br J Cancer.

[25] Huminiecki L, Gorn M, Suchting S, Poulsom R, Bicknell R (2002). Magic roundabout is a new member of the roundabout receptor family that is endothelial specific and expressed at sites of active angiogenesis. Genomics.

[26] Buckanovich RJ, Sasaroli D, O’Brien-Jenkins A, Botbyl J, Hammond R, Katsaros D, Sandaltzopoulos R, Liotta LA, Gimotty PA, Coukos G (2007). Tumor vascular proteins as biomarkers in ovarian cancer. J Clin Oncol.

[27] Jones DT, Lechertier T, Mitter R, Herbert JM, Bicknell R, Jones JL, Li JL, Buffa F, Harris AL, Hodivala-Dilke K (2012). Gene expression analysis in human breast cancer associated blood vessels. PLoS One.

[28] Formica V, Palmirotta R, Del Monte G, Savonarola A, Ludovici G, De Marchis ML, Grenga I, Schirru M, Guadagni F, Roselli M (2011). Predictive value of VEGF gene polymorphisms for metastatic colorectal cancer patients receiving first-line treatment including fluorouracil, irinotecan, and bevacizumab. Int J Colorectal Dis.

[29] Hurwitz HI, Douglas PS, Middleton JP, Sledge GW, Johnson DH, Reardon DA, Chen D, Rosen O (2013). Analysis of early hypertension and clinical outcome with bevacizumab: results from seven phase III studies. Oncologist.

[30] Pentheroudakis G, Kotoula V, Fountzilas E, Kouvatseas G, Basdanis G, Xanthakis I, Makatsoris T, Charalambous E, Papamichael D, Samantas E, Papakostas P, Bafaloukos D, Razis E (2014). A study of gene expression markers for predictive significance for bevacizumab benefit in patients with metastatic colon cancer: a translational research study of the Hellenic Cooperative Oncology Group (HeCOG). BMC Cancer.

[31] Van Cutsem E, de Haas S, Kang YK, Ohtsu A, Tebbutt NC, Ming Xu J, Peng Yong W, Langer B, Delmar P, Scherer SJ, Shah MA (2012). Bevacizumab in combination with chemotherapy as first-line therapy in advanced gastric cancer: a biomarker evaluation from the AVAGAST randomized phase III trial. J Clin Oncol.

[32] Cremolini C, Loupakis F, Bocci G, Falcone A (2014). Biomarkers and response to bevacizumab--letter. Clin Cancer Res.

[33] Bates DO, Catalano PJ, Symonds KE, Varey AH, Ramani P, O’Dwyer PJ, Giantonio BJ, Meropol NJ, Benson AB, Harper SJ (2012). Association between VEGF splice isoforms and progression-free survival in metastatic colorectal cancer patients treated with bevacizumab. Clin Cancer Res.

[34] Jubb AM, Miller KD, Rugo HS, Harris AL, Chen D, Reimann JD, Cobleigh MA, Schmidt M, Langmuir VK, Hillan KJ, Chen DS, Koeppen H (2011). Impact of exploratory biomarkers on the treatment effect of bevacizumab in metastatic breast cancer. Clin Cancer Res.

[35] van Beijnum JR, Dings RP, van der Linden E, Zwaans BM, Ramaekers FC, Mayo KH, Griffioen AW (2006). Gene expression of tumor angiogenesis dissected: specific targeting of colon cancer angiogenic vasculature. Blood.

[36] Parker BS, Argani P, Cook BP, Liangfeng H, Chartrand SD, Zhang M, Saha S, Bardelli A, Jiang Y, St Martin TB, Nacht M, Teicher BA, Klinger KW (2004). Alterations in vascular gene expression in invasive breast carcinoma. Cancer Res.

[37] Madden SL, Cook BP, Nacht M, Weber WD, Callahan MR, Jiang Y, Dufault MR, Zhang X, Zhang W, Walter-Yohrling J, Rouleau C, Akmaev VR, Wang CJ (2004). Vascular gene expression in nonneoplastic and malignant brain. Am J Pathol.

[38] St Croix B, Rago C, Velculescu V, Traverso G, Romans KE, Montgomery E, Lal A, Riggins GJ, Lengauer C, Vogelstein B, Kinzler KW (2000). Genes expressed in human tumor endothelium. Science.

[39] Lu C, Bonome T, Li Y, Kamat AA, Han LY, Schmandt R, Coleman RL, Gershenson DM, Jaffe RB, Birrer MJ, Sood AK (2007). Gene alterations identified by expression profiling in tumor-associated endothelial cells from invasive ovarian carcinoma. Cancer Res.

[40] Bhati R, Patterson C, Livasy CA, Fan C, Ketelsen D, Hu Z, Reynolds E, Tanner C, Moore DT, Gabrielli F, Perou CM, Klauber-DeMore N (2008). Molecular characterization of human breast tumor vascular cells. Am J Pathol.

[41] Pen A, Moreno MJ, Martin J, Stanimirovic DB (2007). Molecular markers of extracellular matrix remodeling in glioblastoma vessels: microarray study of laser-captured glioblastoma vessels. Glia.

[42] Picault FX, Chaves-Almagro C, Projetti F, Prats H, Masri B, Audigier Y (2014). Tumour co-expression of apelin and its receptor is the basis of an autocrine loop involved in the growth of colon adenocarcinomas. Eur J Cancer.

[43] Kalin RE, Kretz MP, Meyer AM, Kispert A, Heppner FL, Brandli AW (2007). Paracrine and autocrine mechanisms of apelin signaling govern embryonic and tumor angiogenesis. Dev Biol.

[44] Cox CM, D’Agostino SL, Miller MK, Heimark RL, Krieg PA (2006). Apelin, the ligand for the endothelial G-protein-coupled receptor, APJ, is a potent angiogenic factor required for normal vascular development of the frog embryo. Dev Biol.

[45] Kasai A, Shintani N, Oda M, Kakuda M, Hashimoto H, Matsuda T, Hinuma S, Baba A (2004). Apelin is a novel angiogenic factor in retinal endothelial cells. Biochem Biophys Res Commun.

[46] Masri B, Morin N, Cornu M, Knibiehler B, Audigier Y (2004). Apelin (65-77) activates p70 S6 kinase and is mitogenic for umbilical endothelial cells. FASEB J.

[47] Masri B, Morin N, Pedebernade L, Knibiehler B, Audigier Y (2006). The apelin receptor is coupled to Gi1 or Gi2 protein and is differentially desensitized by apelin fragments. J Biol Chem.

[48] Sorli SC, Le Gonidec S, Knibiehler B, Audigier Y (2007). Apelin is a potent activator of tumour neoangiogenesis. Oncogene.

[49] Carpene C, Dray C, Attane C, Valet P, Portillo MP, Churruca I, Milagro FI, Castan-Laurell I (2007). Expanding role for the apelin/APJ system in physiopathology. J Physiol Biochem.

[50] Liu Q, Hu T, He L, Huang X, Tian X, Zhang H, He L, Pu W, Zhang L, Sun H, Fang J, Yu Y, Duan S (2015). Genetic targeting of sprouting angiogenesis using Apln-CreER. Nat Commun.

[51] Kidoya H, Naito H, Takakura N (2010). Apelin induces enlarged and nonleaky blood vessels for functional recovery from ischemia. Blood.

[52] Kidoya H, Ueno M, Yamada Y, Mochizuki N, Nakata M, Yano T, Fujii R, Takakura N (2008). Spatial and temporal role of the apelin/APJ system in the caliber size regulation of blood vessels during angiogenesis. EMBO J.

[53] Kasai A, Shintani N, Kato H, Matsuda S, Gomi F, Haba R, Hashimoto H, Kakuda M, Tano Y, Baba A (2008). Retardation of retinal vascular development in apelin-deficient mice. Arterioscler Thromb Vasc Biol.

[54] Heo K, Kim YH, Sung HJ, Li HY, Yoo CW, Kim JY, Park JY, Lee UL, Nam BH, Kim EO, Kim SY, Lee SH, Park JB (2012). Hypoxia-induced up-regulation of apelin is associated with a poor prognosis in oral squamous cell carcinoma patients. Oral Oncol.

[55] Berta J, Kenessey I, Dobos J, Tovari J, Klepetko W, Jan Ankersmit H, Hegedus B, Renyi-Vamos F, Varga J, Lorincz Z, Paku S, Ostoros G, Rozsas A (2010). Apelin expression in human non-small cell lung cancer: role in angiogenesis and prognosis. J Thorac Oncol.

[56] Lacquaniti A, Altavilla G, Picone A, Donato V, Chirico V, Mondello P, Aloisi C, Marabello G, Loddo S, Buemi A, Lorenzano G, Buemi M (2015). Apelin beyond kidney failure and hyponatremia: a useful biomarker for cancer disease progression evaluation. Clin Exp Med.

[57] Zhang L, Takara K, Yamakawa D, Kidoya H, Takakura N (2016). Apelin as a marker for monitoring the tumor vessel normalization window during antiangiogenic therapy. Cancer Sci.

[58] Rayalam S, Della-Fera MA, Krieg PA, Cox CM, Robins A, Baile CA (2008). A putative role for apelin in the etiology of obesity. Biochem Biophys Res Commun.

[59] Betge J, Barat A, Murphy V, Hielscher T, Van Grieken NC, Belle S, Zhan T, Hartel N, Kripp M, Bacon O, Cordes M, Kay EW, Verheul HM (2016). Outcome of colorectal cancer patients treated with combination bevacizumab therapy: a pooled retrospective analysis of three European cohorts from the Angiopredict initiative. Digestion.

[60] Martens-de Kemp SR, Brink A, Stigter-van Walsum M, Damen JM, Rustenburg F, Wu T, van Wieringen WN, Schuurhuis GJ, Braakhuis BJ, Slijper M, Brakenhoff RH (2013). CD98 marks a subpopulation of head and neck squamous cell carcinoma cells with stem cell properties. Stem Cell Res.

[61] Rexhepaj E, Brennan DJ, Holloway P, Kay EW, McCann AH, Landberg G, Duffy MJ, Jirstrom K, Gallagher WM (2008). Novel image analysis approach for quantifying expression of nuclear proteins assessed by immunohistochemistry: application to measurement of oestrogen and progesterone receptor levels in breast cancer. Breast Cancer Res.

[62] Huber W, von Heydebreck A, Sultmann H, Poustka A, Vingron M (2002). Variance stabilization applied to microarray data calibration and to the quantification of differential expression. Bioinformatics.

[63] Ritchie ME, Phipson B, Wu D, Hu Y, Law CW, Shi W, Smyth GK (2015). limma powers differential expression analyses for RNA-sequencing and microarray studies. Nucleic Acids Res.

[64] Abe M, Sato Y (2001). cDNA microarray analysis of the gene expression profile of VEGF-activated human umbilical vein endothelial cells. Angiogenesis.

[65] Schoenfeld J, Lessan K, Johnson NA, Charnock-Jones DS, Evans A, Vourvouhaki E, Scott L, Stephens R, Freeman TC, Saidi SA, Tom B, Weston GC, Rogers P (2004). Bioinformatic analysis of primary endothelial cell gene array data illustrated by the analysis of transcriptome changes in endothelial cells exposed to VEGF-A, PlGF. Angiogenesis.

