# Supplementary material for: Apelin: A putative novel predictive biomarker for bevacizumab response in colorectal cancer
Source: Oncotarget. 2017 Apr 21;8(26):42949–61. doi: 10.18632/oncotarget.17306 (PMC5522118; doi:10.18632/oncotarget.17306)
Supplement: Supplementary file 1 [file oncotarget-08-42949-s001.pdf]

# Apelin: A putative novel predictive biomarker for bevacizumab response in colorectal cancer

## SUPPLEMENTARY MATERIALS

## SUPPLEMENTARY FIGURE AND TABLES

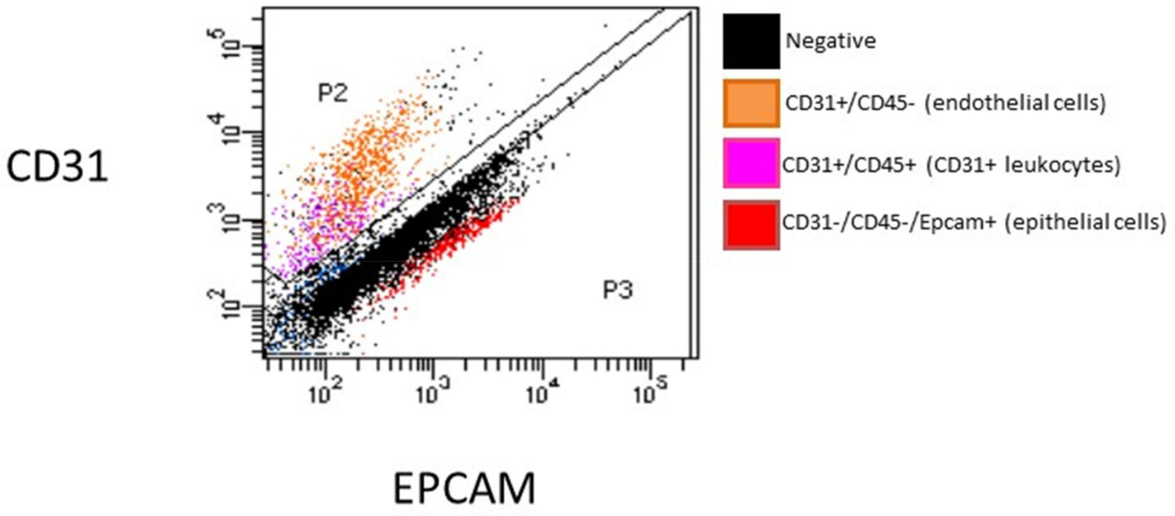


**Supplementary Figure 1: FACS plot showing different populations that were separated during sort procedure.** EPCAM (Fitc) and CD31 (PE) are indicated on the x- and y-axis, respectively. The endothelial cells are depicted in orange, which were separated from CD31+ leukocytes (pink) based on CD45 expression. Epithelial cells (EPCAM+CD45-) are depicted in red and the black cloud mainly indicates autofluorescent cells.

**Supplementary Table 1: Gene sets used to execute GSEA**

| GSEA data sets used for comparison | explanation |
|---|---|
| All 50 HALLMARK gene sets | Specific well-defined biological states or processes |
| BIOCARTA_VEGF_PATHWAY | Biocarta VEGF pathway gene set |
| ABE_VEGFA_TARGETS | Gene sets published by Abe et al, which reflects genes most profoundly induced in HUVEC cells by VEGFA treatment for max. 24 hours |
| ABE_VEGFA_TARGETS_30MIN | Gene sets published by Abe et al, which reflect genes most profoundly induced in HUVEC cells by VEGFA treatment for max 30 min. |
| ABE_VEGFA_TARGETS_2HRS | Gene sets published by Abe et al, which reflect genes upregulated in HUVEC cells by 2 hour VEGFA treatment |
| VEGF_A_UP_V1_UP | Gene sets published by Schoenfeld et al, which are the 200 most upregulated genes in HUVEC cells by treatment with VEGFA |
| VEGF_A_UP_V1_DN | Gene sets published by Schoenfeld et al, which are the 200 most downregulated genes in HUVEC cells by treatment with VEGFA |

**Supplementary Table 2: Top-10 "Hallmark gene sets" of GSEA**

| NAME | SIZE | NES | FDR q-val | RANK AT MAX | LEADING EDGE |
|---|---|---|---|---|---|
| HALLMARK_DNA_REPAIR | 143 | 1.49 | 0.13 | 3662 | Tags=41%, list=19%, signal=51% |
| HALLMARK_EPITHELIAL_ MESENCHYMAL_TRANSITION | 199 | 1.46 | 0.14 | 2097 | Tags=43%, list=11%, signal=48% |
| HALLMARK_ANGIOGENESIS | 36 | 1.49 | 0.15 | 3089 | Tags=58%, list=16%, signal=70% |
| HALLMARK_TGF_BETA_SIGNALING | 54 | 1.46 | 0.15 | 3744 | Tags=54%, list=20%, signal=67% |
| HALLMARK_COAGULATION | 135 | 1.44 | 0.16 | 1130 | Tags=30%, list=6%, signal=32% |
| HALLMARK_APICAL_SURFACE | 44 | 1.42 | 0.16 | 2226 | Tags=32%, list=12%, signal=36% |
| HALLMARK_MYC_TARGETS_V1 | 198 | 1.49 | 0.18 | 4180 | Tags=61%, list=22%, signal=77% |
| HALLMARK_G2M_CHECKPOINT | 200 | 1.39 | 0.20 | 3922 | Tags=52%, list=21%, signal=65% |
| HALLMARK_MITOTIC_SPINDLE | 199 | 1.50 | 0.23 | 2871 | Tags=37%, list=15%, signal=43% |
| HALLMARK_E2F_TARGETS | 198 | 1.36 | 0.23 | 3938 | Tags=51%, list=21%, signal=64% |

The top-10 gene sets are ranked on FDR. "Name" indicates the biological states or process represented by the gene set, "size" shows the number of genes included in the gene set, NES is the nominal enrichment score, FDR q-val is the *p*-value corrected for false discovery, "rank at max" means the position in the ranked list at which the maximum enrichment score occurred and the "leading edge" informs about the percentage of gene hits (tags) or genes in the ranked list (list) before the peak in the running enrichment score as well as the enrichment strength (signal).
